# Developmental brain lipidomics is influenced by postnatal chlorpyrifos exposure and *APOE* genetic background in mice

**DOI:** 10.1007/s00204-023-03555-8

**Published:** 2023-07-13

**Authors:** Laia Guardia-Escote, Judit Biosca-Brull, Maria Cabré, Jordi Blanco, Mikaela Mladenova-Koleva, Pia Basaure, Cristian Pérez-Fernández, Fernando Sánchez-Santed, José L. Domingo, Maria Teresa Colomina

**Affiliations:** 1 Research Group in Neurobehavior and Health (NEUROLAB), Universitat Rovira i Virgili, Tarragona, Spain; 2 Department of Psychology and Research Center for Behavior Assessment (CRAMC), Universitat Rovira i Virgili, Tarragona, Spain; 3 Laboratory of Toxicology and Environmental Health (TECNATOX), Universitat Rovira i Virgili, Reus, Spain; 4 Department of Biochemistry and Biotechnology, Universitat Rovira i Virgili, Tarragona, Spain; 5 Department of Basic Medical Sciences, Universitat Rovira i Virgili, Reus, Spain; 6 Department of Psychology, Health Research Center (CEINSA), Almería University, Almería, Spain

**Keywords:** Lipidomics, *APOE*, Chlorpyrifos, Brain development

## Abstract

Lipids are a major component of the brain, and are involved in structural and neurodevelopmental processes such as neurogenesis, synaptogenesis and signaling. Apolipoprotein E (apoE) is the main lipoprotein involved in lipid transport in the brain. The apoE isoforms can determine vulnerability to the toxic effects of the pesticide chlorpyrifos (CPF), which can interfere with normal neurodevelopment. We aimed to study the effects of postnatal exposure to CPF and of the *APOE* genotype on the lipid composition of the brain at early ages. For it, we used apoE3 and apoE4 targeted-replacement (TR) male mice, as well as wild-type C57BL/6. The mice were orally exposed to 1 mg/kg/day of CPF on postnatal days 10–15 and, four hours after the treatment, we obtained samples to assess the cerebral lipid composition. Differences between *APOE* genotypes were found in the cerebral lipid profile in the postnatal period. ApoE4-TR mice exhibited higher lipid concentrations compared to the other groups in most of the cases. CPF exposure led to a decrease in cholesteryl ester and triglyceride concentrations, while modulating the levels of phosphatidylcholine species based on the apoE isoform. Specifically, CPF treatment decreased the concentration of some species of this lipid (PC30:0, PC31:0, PC32:2, PC36:5, PC40:4 and PC40:5) in C57BL/6 mice exposed to CPF, increased (PC31:0 and PC37:6) in apoE3-TR exposed mice while exposed apoE4-TR mice remained unaltered. These results provide further insights into the lipid composition of the brain at early ages, and how it can be modulated by environmental and genetic factors.

## Introduction

The brain is an organ that is extremely rich in lipids, which play a critical role in neurodevelopment. During the first postnatal weeks, important events involving lipids, such as myelin sheath formation or membrane maturation, take place in the brain. Most lipid families undergo several changes in order to play this role, with a rapid increase in their brain concentrations after birth. For instance, cholesterol, a key component of myelin and involved in synaptogenesis, is highly synthesized during the first postnatal weeks while sphingomyelin, present in the myelin sheath and cell membranes, undergoes a significant increase during the first years of life (Dawson 2015). Any alteration in these processes can cause permanent impairments and lead to neurodevelopmental disorders. In human adults, lipids constitute 50–60% of the dry weight of the brain and present considerable structural diversity. For example, they can differ in the carbon chain length or in the saturation degree. It has been reported that different cell types and different regions of the brain have their own unique lipid composition. It defines their specific functions in the brain: namely, they provide cell membranes with structural integrity, store energy or promote molecular signaling (Fitzner et al. 2020; Ooi et al. 2021). Alterations in the integrity of lipid composition have been related with aging and brain dysfunction, including the onset of neurodegenerative diseases. These include Alzheimer’s disease (AD), Parkinson’s disease and Huntington’s disease, among others (Vance and Hayashi 2010). AD, one of the most studied, has been associated with abnormal levels of cholesterol, sphingolipids, phospholipids and glycerolipids in the brain (Wong et al. 2017).

Lipid transport and homeostasis in the brain are regulated by lipoproteins, with apolipoprotein E (apoE) being one of their primary constituents in the central nervous system (CNS). The *APOE* gene is a polymorphic gene that has three main isoforms in humans—apoE2, apoE3 and apoE4—each of which has different affinities for lipoproteins. More specifically, apoE3 binds to small HDL, while apoE4 binds to large VLDL and chylomicron remnants (Huang and Mahley 2014; Mahley 2016). In the CNS, apoE is synthesized mostly by astrocytes, provided that liver-synthesized apoE cannot cross the blood–brain barrier (BBB) (Liu et al. 2012). Indeed, the role of apoE is essential for correct brain functioning. ApoE helps distribute cholesterol and phospholipids throughout the brain to cover for neurite outgrowth, neurodevelopment, and repair and remodeling in case of brain injury (Han 2004; Vance and Hayashi 2010). Functional differences have been reported between apoE isoforms, which confer different vulnerabilities to some diseases (such as AD) or environmental toxicants (Roses 1996; Engstrom et al. 2017).

Chlorpyrifos (CPF) is an organophosphate (OP) pesticide that has been commonly used for fruit and crop protection. In recent decades, some restrictions have been placed on its use (Nandi et al. 2022). In general, pesticide use is a public health concern because the general population is exposed to chronic low-doses of pesticides in the diet. Children, in particular, are an important risk group. This is because their body weight is lower and they present lower levels of paraoxonase 1 (PON1) enzymes, an esterase that can hydrolyze the active metabolites of many OP pesticides, such as CPF (Costa et al. 2013). CPF exerts its toxicity by inhibiting the enzyme Acetylcholinesterase (AChE), thus directly attacking the cholinergic system in the brain (Pope 1999). However, secondary targets have also been reported, including specific lipases involved in lipid homeostasis (Casida et al. 2008). Developmental exposure to CPF has been widely studied because young animals are more sensitive to toxic exposure than adults (Pope 1999; Moser 2000). In fact, a significant correlation has been reported between developmental exposure to CPF and postnatal impairments in locomotor activity, cognition, social discrimination, anxiety response and working memory (Ricceri et al. 2006; De Felice et al. 2014; Burke et al. 2017).

The present investigation was aimed at assessing the effects of postnatal CPF exposure and the influence of *APOE* genetic background on the lipid composition of the brain at early ages. *Omic* technologies have provided new tools for studying these effects on lipid composition (Hussain et al. 2019). This study was conducted in apoE targeted replacement (TR) mice expressing the human isoform for the *ε3* and *ε4* alleles, which were exposed to CPF during the postnatal period between days 10 and 15. To the best of our knowledge, this is the first study in which the lipid composition of the brain after CPF exposure has been assessed at such a young age.

## Material and methods

### Animals and care

Male apoE3-TR and apoE4-TR mice (Taconic Europe, Lille Skensved, Denmark) and wild type C57BL/6 J (Charles River, L’Arbresle, France) were used in this study. The apoE-TR model has a C57BL/6NTac background and presents the human *ε3* or *ε4* alleles instead of the murine gene (Sullivan et al. 1997). Mice of the same genotype were mated and then we monitored the body weight of females. Pregnant females were maintained in individual cages and the day of delivery was considered postnatal day (PND) 0. All animals had access to fresh water and a normal chow diet (SAFE A04 diet, supplied by Panlab, Barcelona, Spain). They were kept under controlled conditions (22 ± 2 °C and 50 ± 10% humidity) on an automatic light/dark cycle every 12 h (lights on at 8 am). The use of animals and the experimental protocol were approved by the Animal Care and Use Committee of the Rovira i Virgili University (Tarragona, Spain) and at all times complied with the Spanish Royal Decree 53/2013 on the protection of experimental animals, and the European Communities Council Directive (2010/63/EU).

### Chemical compounds and treatment

CPF [0,0-diethyl O-(3,5,6-trichloropyridin-2-yl) phosphorothioate], with a purity of 99.5%, was purchased from Sigma-Aldrich Co. LLC. (Madrid, Spain). It was dissolved in the vehicle corn oil and adjusted so that 1 mg/kg was administered in 1 μL/g of body weight. The animals were divided into two groups: the CPF-treated group was orally administered the pesticide with a micropipette, whereas the control group was administered the vehicle. The treatment period lasted from PND 10 to PND 15, both inclusive. A total of 36 males were included in the study, distributed in six groups. However, an outlier was detected during data processing and, consequently, it was eliminated from the statistical analysis. Therefore, the final number of animals was 35, distributed as follows: Control C57BL/6 (n = 6), CPF-treated C57BL/6 (n = 6), Control apoE3 (n = 6), CPF-treated apoE3 (n = 6), Control apoE4 (n = 5) and CPF-treated apoE4 (n = 6).

### Sacrifice and sampling

Mice were euthanized on PND 15, 4 h after exposure to the last dose of CPF. Previous studies have revealed an inhibitory effect on AChE activity in plasma but no differences in the AChE activity in the forebrain 4 h after exposure to CPF (Basaure et al. 2018). The animals were deeply anesthetized with isoflurane before being euthanized by decapitation. Brain samples were obtained, immediately snap frozen in liquid nitrogen and then stored at – 80 °C for subsequent analysis.

### Lipidomic profile

The lipidomic profile was determined in an external laboratory, the Centre for Omic Sciences (COS) in Reus, Spain. Whole brain samples were homogenized, and 5 mg of the tissue was extracted with chloroform:methanol. The lower phase was recovered after centrifuging with water and NaCl (0.9%), and reconstituted with methanol:methyl-tert-butyl ether. Analysis was performed by UHPLC-qTOF (model 6550 of Agilent, USA) in positive electrospray ionization mode. Chromatographic gradient elution with a ternary mobile phase containing water, methanol and 2-propanol with 10 mM ammonium formate and 0.1% formic acid was performed using a C18 column (Kinetex EVO C18 Column, 2.6 μm, 2.1 mm × 100 mm) as a stationary phase, allowing the sequential elution of the more hydrophobic lipids. The lipid species were identified by matching their accurate mass and tandem mass spectrum to Metlin-PCDL from Agilent and by matching the chromatographic behavior of pure standards for each family of lipids. Then, lipids were semi-quantified depending on their family similarity by internal standard calibration curves using pure chemical standards. Finally, concentration was calculated by normalization with the original tissue weight for each brain sample.

### Statistical analysis

Data were analyzed with the SPSS 26.0 software (IBM Corp, Chicago, USA). The lipidomic profile for each lipid family was plotted as a heatmap with the metabolomics data analysis platform MetaboAnalyst. A two-way analysis of variance (ANOVA) was used to study the effects of *APOE* and CPF exposure on the concentrations of the various lipid species. A one-way ANOVA (group) and a post-hoc Tukey’s test of variance were used to analyze differences between groups. The variance homogeneity was assessed by a Levene test. Statistical significance was set at *p* < 0.05. Results are reported as mean values ± S.E.M.

## Results

### Lipidomics

Lipid distribution in the brain is reported below for each of the lipid families studied. The first number refers to the acyl carbon atoms, and the second to the number of unsaturations.

#### Cholesteryl ester

**Fig. 1:**
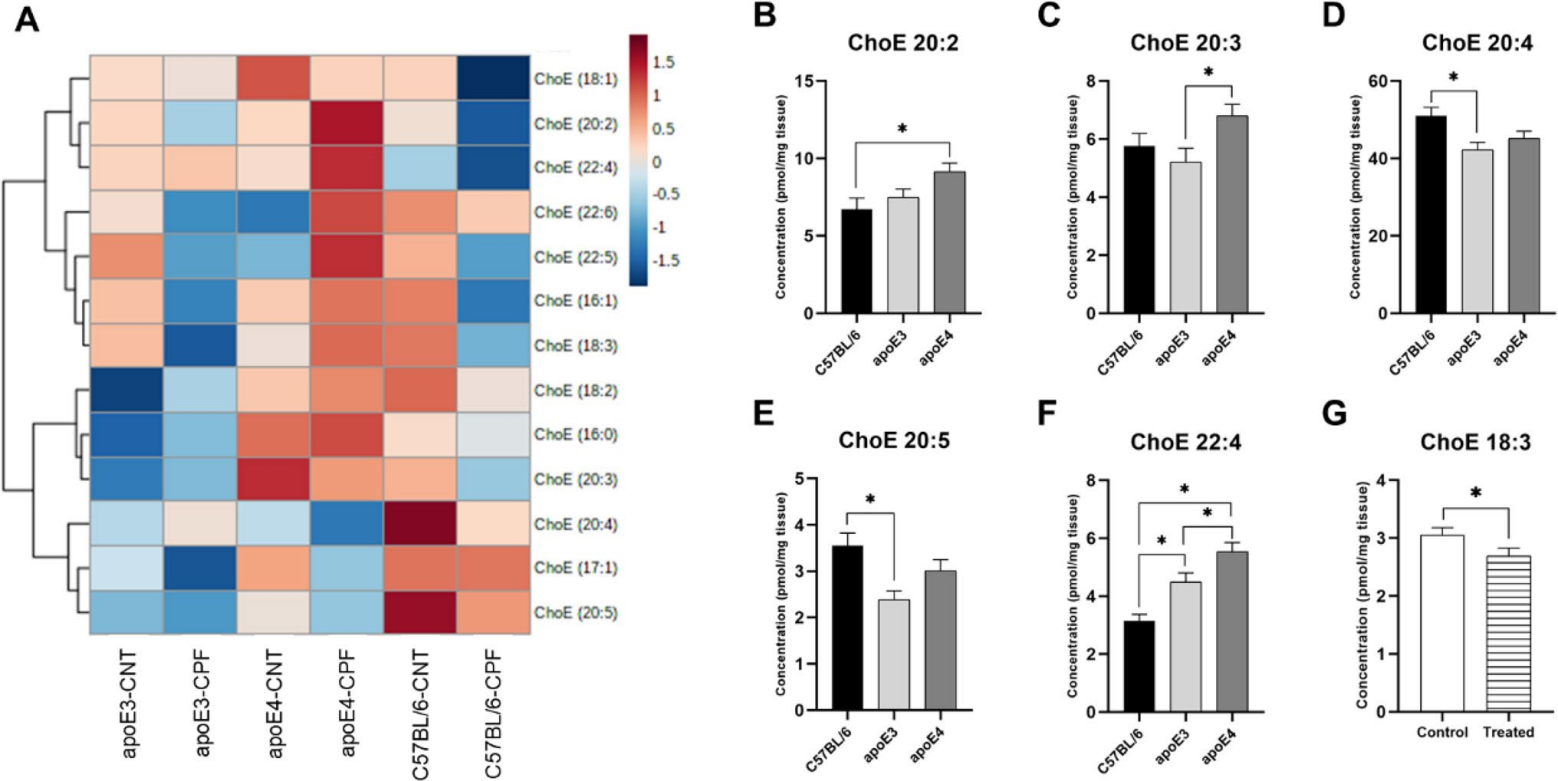
Cholesteryl ester (ChoE) profile in mouse brain. Heatmap of the brain concentration (pmol/mg tissue) of the ChoE species for each experimental group (**A**). ChoE species presenting significant differences between genotypes (**B**–**F**) and treatments (**G**). All groups included six animals except for the apoE4-CNT, which included five. An asterisk (*) indicates significant differences between groups at *p* < 0.05. Abbreviations: CNT control, CPF chlorpyrifos-treated

A total of thirteen cholesteryl ester (ChoE) species were analyzed, as depicted in the heatmap in Fig. 1A. A two-way ANOVA (genotype × treatment) found significant effects of the genotype in several ChoE species, including: ChoE 20:2 [F(2,29) = 4.300, *p* = 0.023], ChoE 20:3 [F(2,29) = 3.332, *p* = 0.050], ChoE 20:4 [F(2,29) = 4.943, *p* = 0.014], ChoE 20:5 [F(2,29) = 6.347, *p* = 0.005] and ChoE 22:4 [F(2,29) = 18.346, *p* < 0.001]. In order to further investigate these differences, we performed a one-way ANOVA (group). *Post-hoc* analysis revealed that apoE4-TR mice presented significantly higher concentrations of ChoE 20:2 and ChoE 20:3 than C57BL/6 and apoE3-TR mice, respectively (Fig. 1B and C). In turn, C57BL/6 mice showed a higher concentration of ChoE 20:4 and ChoE 20:5 than apoE3-TR mice (Fig. 1D and F). ApoE4-TR mice presented the highest concentrations of ChoE 22:4, and C57BL/6 the lowest, both showing significant differences between them, and in comparison to apoE3-TR mice (Fig. 1F).

**Fig. 2:**
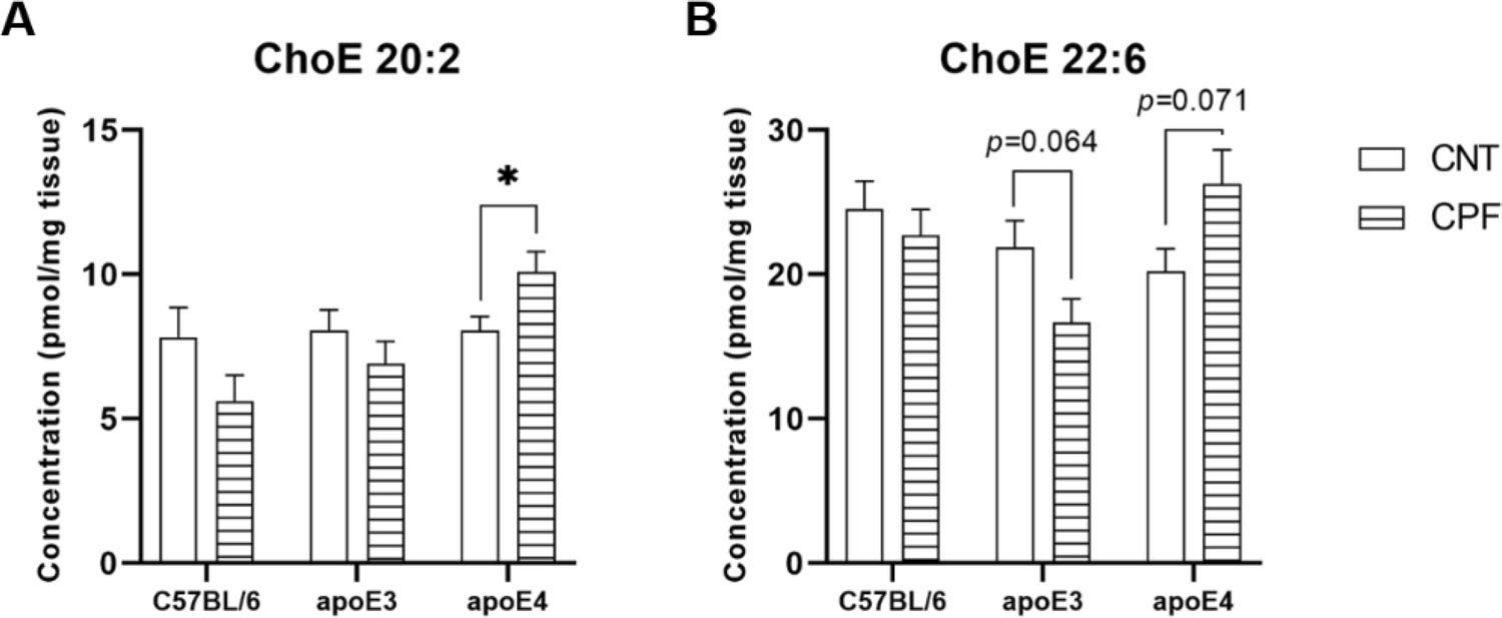
Cholesteryl ester (ChoE) species presenting a significant interaction between genotype and treatment. All groups included six animals except for the apoE4-CNT, which included five. An asterisk (*) indicates significant differences between groups at *p* < 0.05. Abbreviations: CNT control, CPF chlorpyrifos-treated

CPF treatment had a significant effect on ChoE 18:3 [F(1,29) = 4.533, *p* = 0.042], with the CPF-treated group presenting lower concentrations than the control group (Fig. 1G). An interaction between genotype and treatment was also observed in ChoE 20:2 [F(2,29) = 3.640, *p* = 0.039] and ChoE 22:6 [F(2,29) = 4.506, *p* = 0.020]. Further analysis revealed an increase in the CPF-treated apoE4-TR mice in comparison to their control in ChoE 20:2 (*p* = 0.050), as well as a tendency in ChoE 22:6 (*p* = 0.071). A tendency towards a decrease in ChoE 22:6 was observed in the CPF-treated apoE3-TR mice, whereas no significant differences were observed in the C57BL/6 group (Fig. 2A and B).

#### Diglyceride

**Fig. 3:**
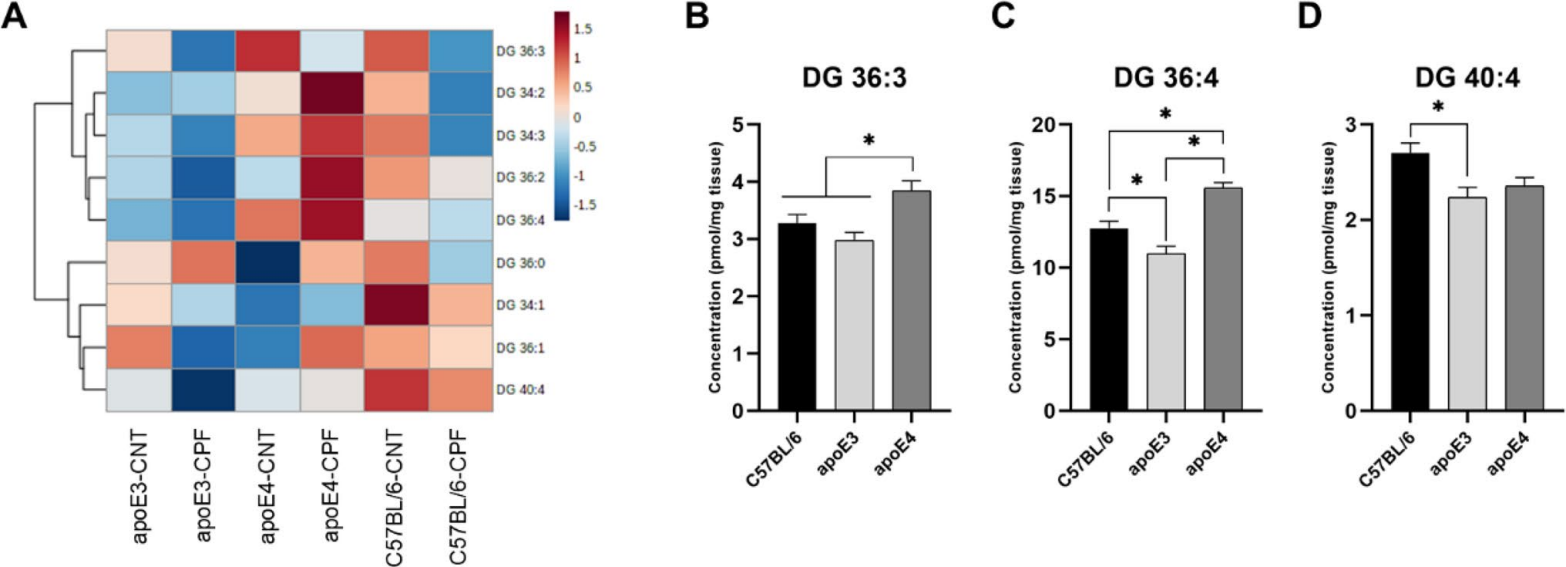
Diglyceride (DG) profile in mouse brain. Heatmap of the brain concentration (pmol/mg tissue) of the DG species for each experimental group (**A**). DG species presenting significant differences between genotype (**B**–**D**). All groups included six animals except for the apoE4-CNT, which included five. An asterisk (*) indicates significant differences between groups at *p* < 0.05. Abbreviations: CNT control, CPF chlorpyrifos-treated

Nine species from the diglyceride (DG) lipid family were analyzed (Fig. 3A). To study them in greater detail we performed a two-way ANOVA (genotype × treatment). Genotype differences were found in the following cases: DG 36:3 [F(2,29) = 8.029, *p* = 0.002], DG 36:4 [F(2,29) = 26.590, *p* < 0.001] and DG 40:4 [F(2,29) = 5.729, *p* = 0.008]. *Post-hoc* analysis found that apoE4-TR mice had higher concentrations of DG 36:3 and DG 36:4 than the other genotypes (Fig. 3B and C). Nonetheless, in DG 36:4, a significant difference between C57BL/6 and apoE3-TR mice was also observed. C57BL/6 mice showed higher DG 40:4 values than apoE3-TR mice (Fig. 3D).

#### Lysophosphocholine

**Fig. 4:**
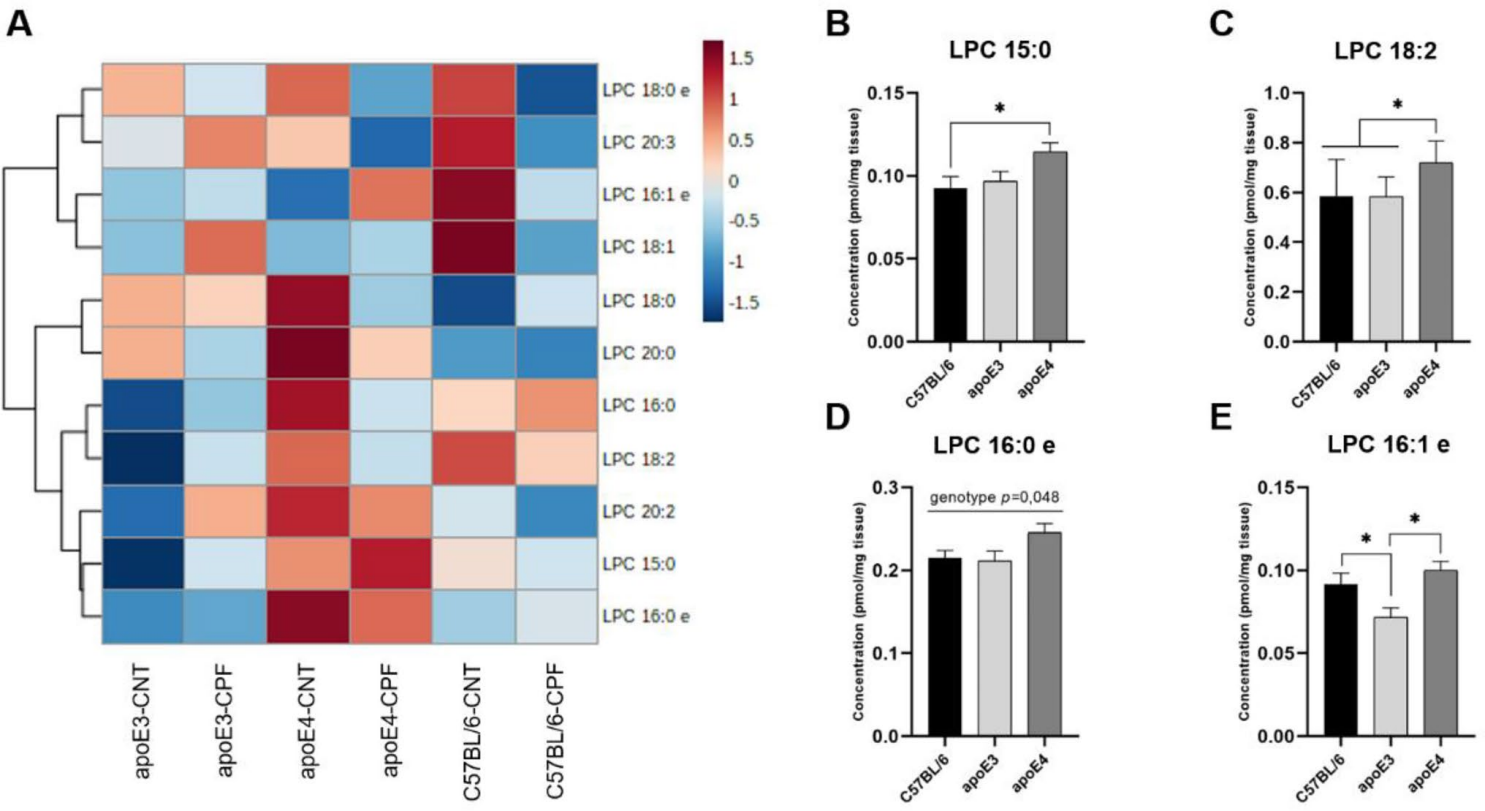
Lysoposphocholine (LPC) profile in mouse brain. Heatmap of the brain concentration (pmol/mg tissue) of the LPC species for each experimental group (**A**). LPC species presenting significant differences between genotypes (**B**–**E**). All groups included six animals except for the apoE4-CNT, which included five. An asterisk (*) indicates significant differences between groups at *p* < 0.05. Abbreviations: CNT control, CPF chlorpyrifos-treated

A total of eleven lysophosphocholine (LPC) species were included to obtain a general screening of their concentration in the brain (Fig. 4A). A two-way ANOVA (genotype x treatment) found genotype effects in LPC 15:0 [F(2,29) = 3.375, *p* = 0.048], LPC 18:2 [F(2,29) = 5.763, *p* = 0.008], LPC 16:0e [F(2,29) = 3.371, *p* = 0.048] and LPC 16:1e [F(2,29) = 5.604, *p* = 0.009]. Further *post-hoc* analysis found higher concentrations of LPC 15:0 and 18:2 in the apoE4-TR group compared to the other genotypes (Fig. 4B and C). In turn, the study of LPC 16:1e found lower concentrations in apoE3-TR mice than in the other groups (Fig. 4E).

#### Phosphatidylcholine

**Fig. 5:**
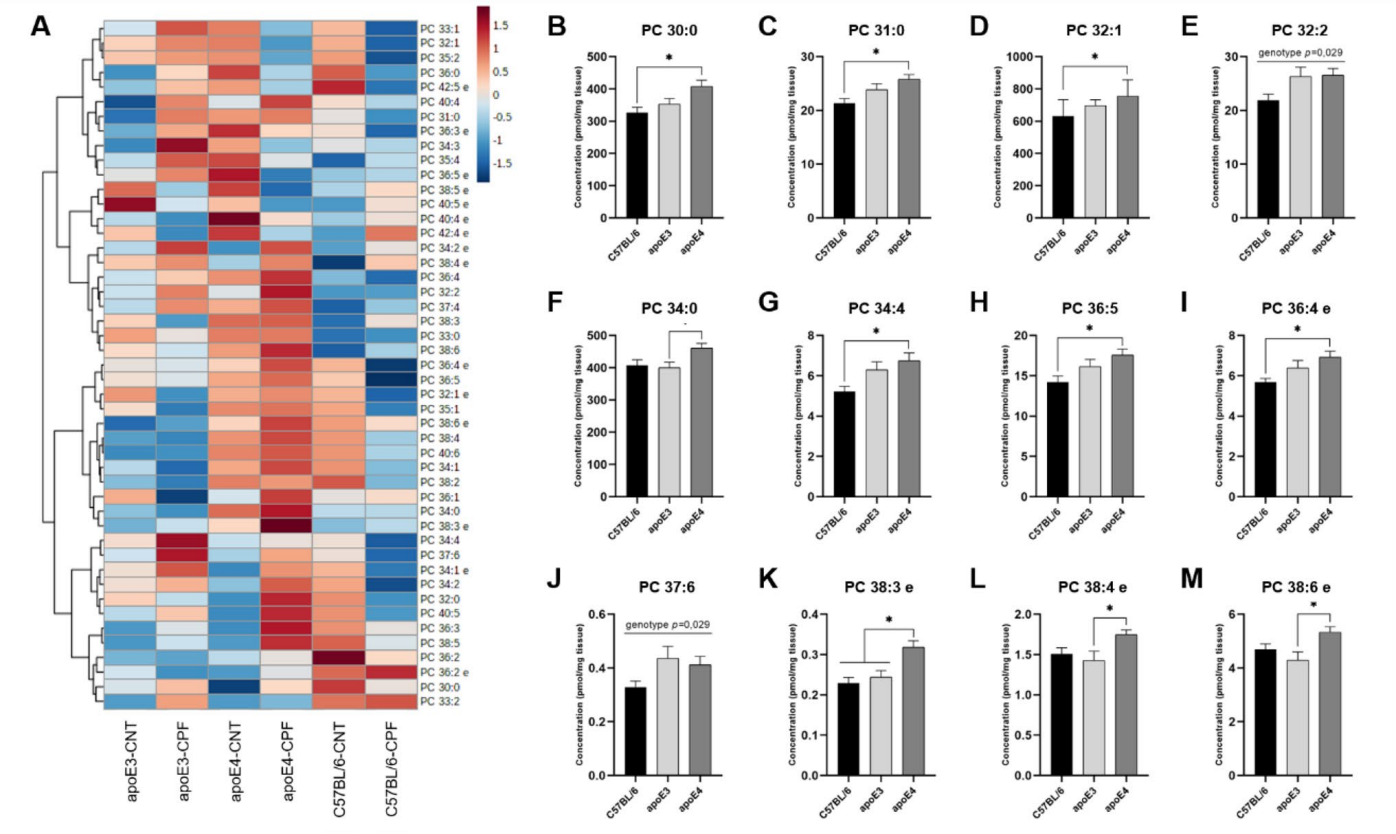
Phosphatidylcholine (PC) profile in mouse brain. Heatmap of the brain concentration (pmol/mg tissue) of the PC species for each experimental group (**A**). PC species presenting significant differences between genotypes (**B**–**M**). All groups included six animals except for the apoE4-CNT, which included five. An asterisk (*) indicates significant differences between groups at *p* < 0.05. Abbreviations: CNT control, CPF chlorpyrifos-treated

The brain concentration of forty-seven different phosphatidylcholine (PC) species was analyzed (Fig. 5A). A two-way ANOVA (genotype x treatment) indicated genotype differences in several PC species, including: PC 30:0 [F(2,29) = 5.620, *p* = 0.009], PC 31:0 [F(2,29) = 6.455, *p* = 0.005], PC 32:1 [F(2,29) = 4.070, *p* = 0.028], PC 32:2 [F(2,29) = 3.995, *p* = 0.029], PC 34:0 [F(2,29) = 4.510, *p* = 0.020], PC 34:4 [F(2,29) = 5.285, *p* = 0.011], PC 36:5 [F(2,29) = 4.846, *p* = 0.015], PC 36:4e [F(2,29) = 4.303, *p* = 0.023], PC 37:6 [F(2,29) = 3.993, *p* = 0.029], PC 38:3e [F(2,29) = 9.097, *p* = 0.001], PC 38:4e [F(2,29) = 3.437, *p* = 0.046] and PC 38:6e [F(2,29) = 4.151, *p* = 0.026]. Further *post-hoc* analysis found the same tendency towards a significantly higher concentration in apoE4-TR mice in comparison to the other genotypes, especially C57BL/6, which showed the lowest concentrations (Fig. 5B–M).

**Fig. 6:**
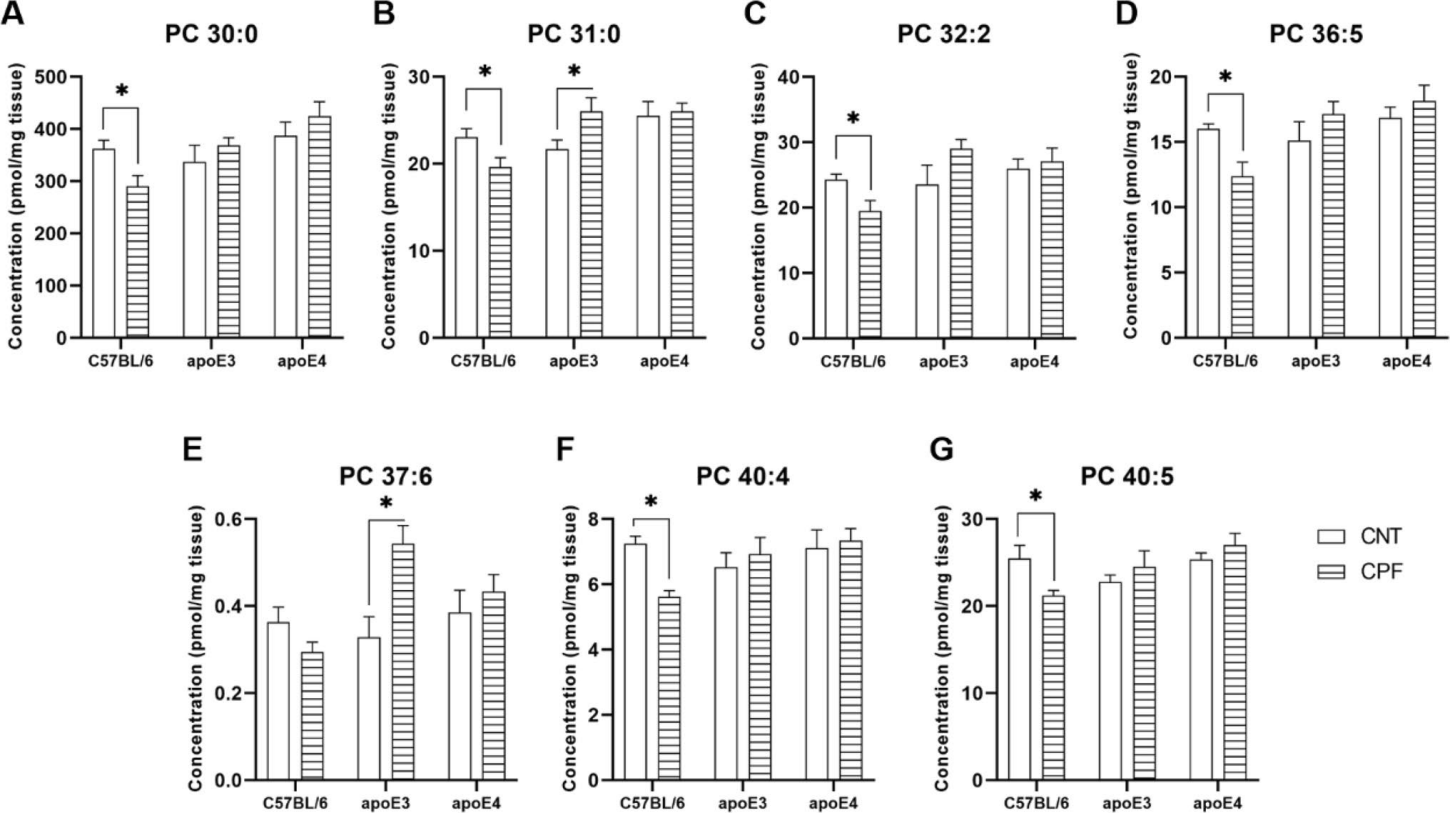
Phosphatidylcholine (PC) species presenting a significant interaction between genotype and treatment. All groups included six animals except for the apoE4-CNT, which included five. An asterisk (*) indicates significant differences between groups at *p* < 0.05. Abbreviations: CNT control, CPF chlorpyrifos-treated

On the other hand, an interaction between genotype and treatment was observed in PC 30:0 [F(2,29) = 3.385, *p* = 0.048], PC 31:0 [F(2,29) = 5.203, *p* = 0.012], PC 32:2 [F(2,29) = 3.967, *p* = 0.030], PC 36:5 [F(2,29) = 4.322, *p* = 0.023], PC 37:6 [F(2,29) = 6.565, *p* = 0.004], PC 40:4 [F(2,29) = 4.010, *p* = 0.029] and PC 40:5 [F(2,29) = 3.872, *p* = 0.032]. Further analysis revealed that the concentrations of PC 30:0 (*p* = 0.021), PC 31:0 (*p* = 0.039), PC 32:2 (*p* = 0.022), PC 36:5 (*p* = 0.010), PC 40:4 (*p* < 0.001) and PC 40:5 (*p* = 0.024) decreased in CPF-treated C57BL/6 mice. Moreover, CPF-treated apoE3-TR mice showed higher levels of PC 31:0 (*p* = 0.045) and PC 37:6 (*p* = 0.006), whereas no significant effect of the treatment was observed on the apoE4-TR mice (Fig. 6A–G).

#### Phosphatidylethanolamines

**Fig. 7:**
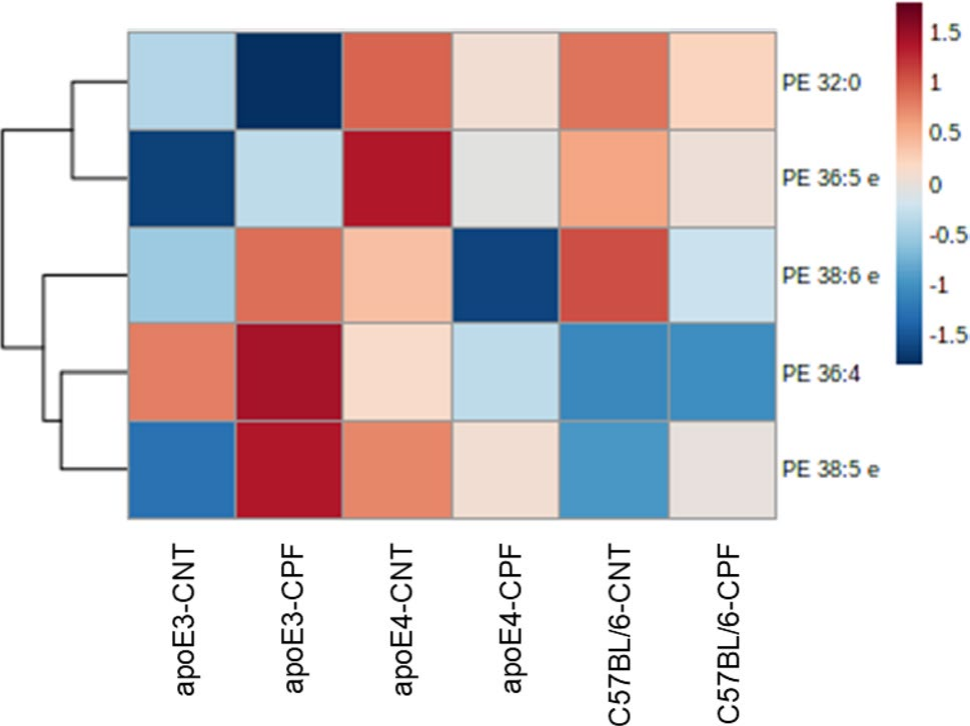
Phosphatidylethanolamine (PE) profiles in mouse brain. Heatmap of the brain concentration (pmol/mg tissue) of the PE species for each experimental group. All groups included six animals except for the apoE4-CNT, which included five. Abbreviations: CNT control, CPF chlorpyrifos-treated

Five phosphatidylethanolamine (PE) species were analyzed in the brain. The heatmap with the concentrations per group is shown in Fig. 7. No significant differences were found between groups.

#### Sphingomyelin

**Fig. 8:**
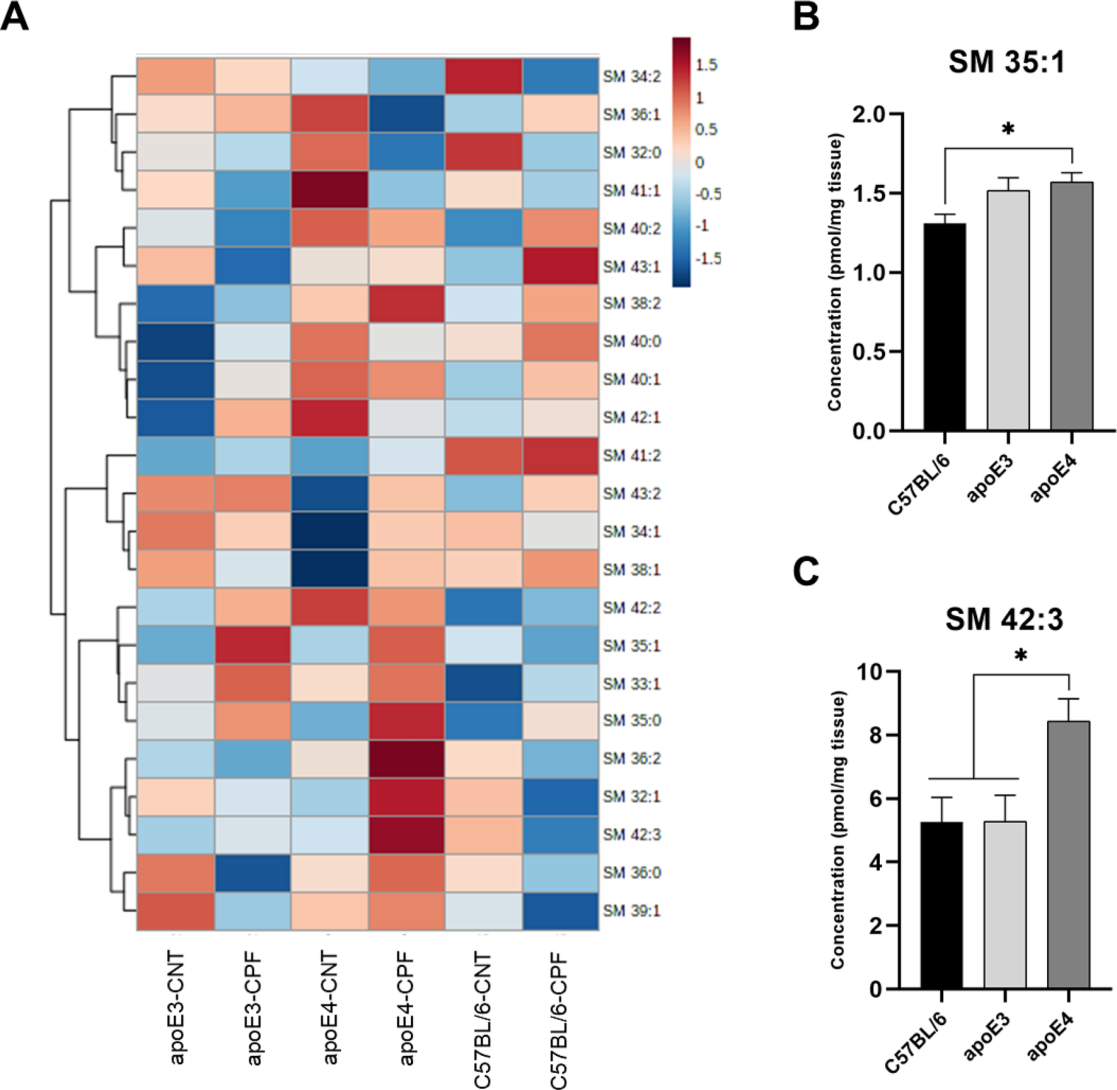
Sphingomyelin (SM) profile in mouse brain. Heatmap of the brain concentration (pmol/mg tissue) of the SM species for each experimental group (**A**). SM species presenting significant differences between genotypes (**B**–**C**). All groups included six animals except for the apoE4-CNT, which included five. An asterisk (*) indicates significant differences between groups at *p* < 0.05. Abbreviations: CNT control, CPF chlorpyrifos-treated

A total of twenty-three sphingomyelin (SM) species were analyzed in the brain (Fig. 8A). To examine the differences between groups, we performed a two-way ANOVA (genotype × treatment). Genotype differences were observed in SM 35:1 [F(2,29) = 4.181, *p* = 0.025] and SM 42:3 [F(2,29) = 5.390, *p* = 0.010], while concentrations were higher in apoE4-TR mice than in C57BL/6 (Fig. 8B) and both C57BL/6 and apoE3-TR groups (Fig. 8C).

#### Triglycerides

**Fig. 9:**
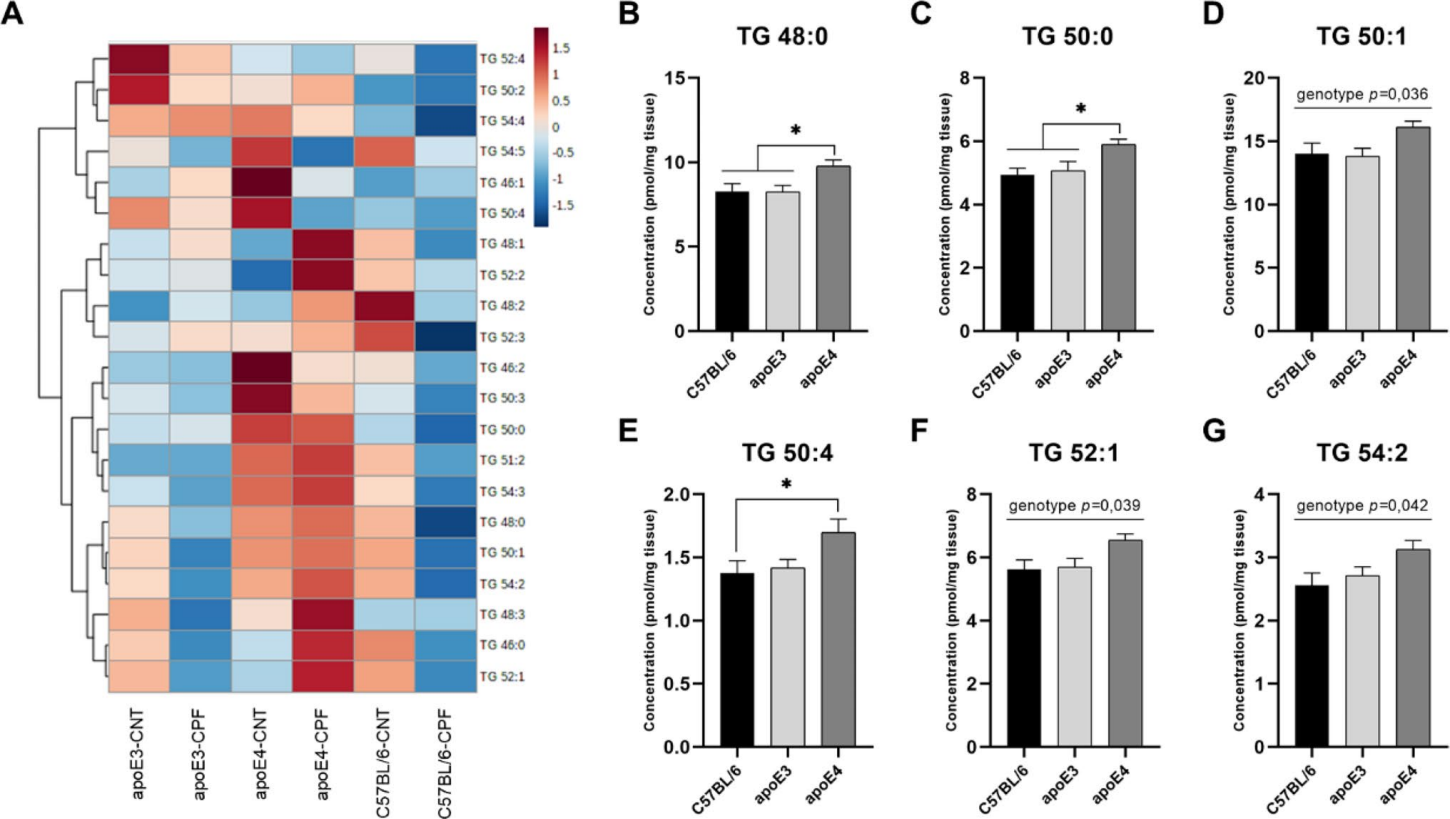
Triglyceride (TG) profiles in mouse brain. Heatmap of the brain concentration (pmol/mg tissue) of the TG species for each experimental group (**A**). TG species presenting significant differences between genotypes (**B**–**G**). All groups included six animals except for the apoE4-CNT, which included five. An asterisk (*) indicates significant differences between groups at *p* < 0.05. Abbreviations: CNT control, CPF chlorpyrifos-treated

Twenty-one triglyceride (TG) species were analyzed for the different groups (Fig. 9A). A two-way ANOVA (genotype × treatment) found a genotype effect in TG 48:0 [F(2,29) = 5.445, *p* = 0.010], TG 50:0 [F(2,29) = 5.599, *p* = 0.009], TG 50:1 [F(2,29) = 3.739, *p* = 0.036], TG 50:4 [F(2,29) = 3.353, *p* = 0.049], TG 52:1 [F(2,29) = 3.629, *p* = 0.039] and TG 54:2 [F(2,29) = 3.539, *p* = 0.042]. Further *post-hoc* analysis found that apoE4-TR mice had higher concentrations of the different TG species than the other genotypes (Fig. 9B–G).

**Fig. 10:**
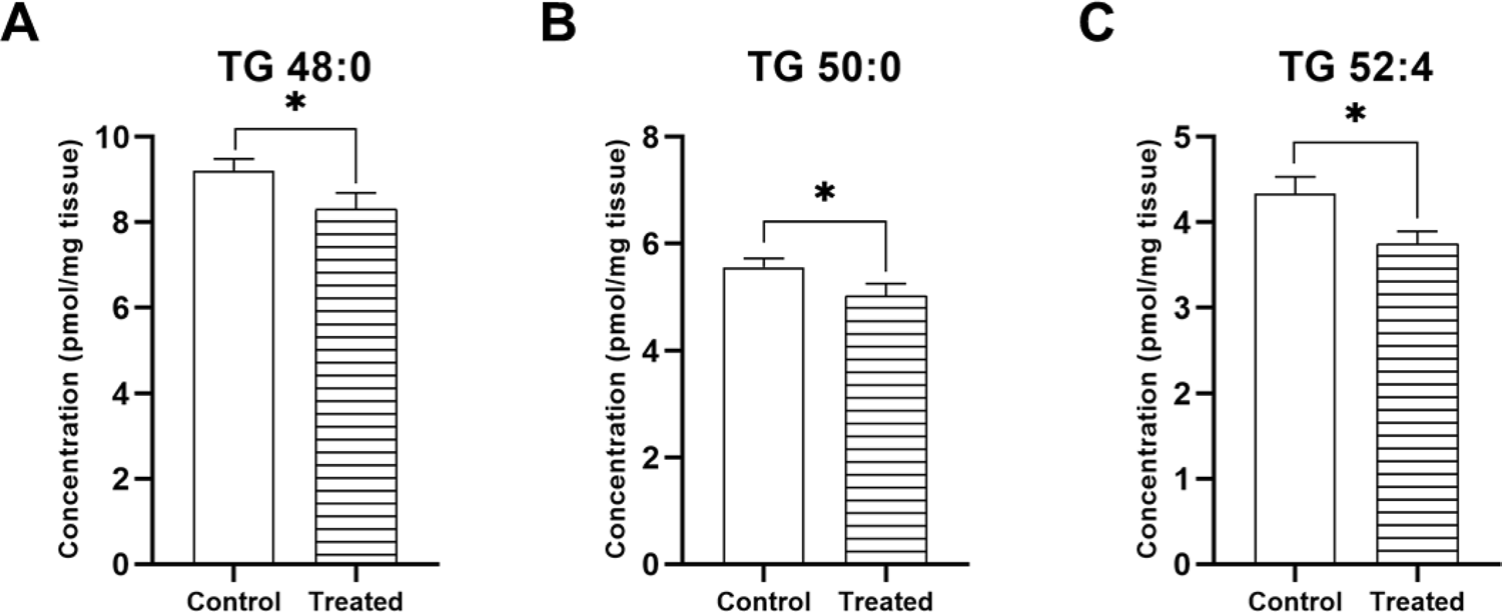
Triglyceride (TG) species presenting significant differences between treatments. All groups included six animals except for the apoE4-CNT, which included five. An asterisk (*) indicates significant differences between groups at *p* < 0.05

Besides, a significant effect of the treatment was found in TG 48:0 [F(1,29) = 4.963, *p* = 0.034], TG 50:0 [F(1,29) = 4.897, *p* = 0.035] and TG 52:4 [F(1,29) = 5.443, *p* = 0.027]. *Post-hoc* analysis found that the CPF-treated group had lower concentrations of TG species than the control group (Fig. 10A–C).

## Discussion

The present study was designed to determine the impact of *APOE* genotype and postnatal exposure to CPF on early differences in the brain lipid profile of mice. The concentration of the different lipid species was studied on PND 15, 4 h after exposure to CPF. Almost all the groups analyzed showed differences related to the genetic background, and CPF exposure had effects in both apoE-TR and C57BL/6 mice, yet sometimes in the opposite direction. Therefore, the current results showed a complex regulation of the lipid profile in the brain, influenced by *APOE* genotype and postnatal CPF exposure, as well as the interaction between them.

In our laboratory, we have extensively investigated the differences between *APOE* genotypes, including differences in learning and memory (Basaure et al. 2019; Guardia-Escote et al. 2019), attention and inhibitory control (Reverte et al. 2016), metabolism (Peris-Sampedro et al. 2018), gut microbiota composition (Guardia-Escote et al. 2020) and epigenetic regulation (Guardia-Escote et al. 2021). The role of apoE in lipid metabolism and transport in the brain is well-characterized, as is the isoform-dependent binding affinity for the various lipoproteins (Raber et al. 2002; Huang and Mahley 2014). Our results showed a significant effect of the *APOE* genotype on the concentrations of several lipid species from almost all the studied families: namely, ChoE, DG, LPC, PC, SM and TG. A previous study including apoE^+/+^, apoE^−/−^ and human apoE-TR mice at 12 months of age found no differences in lipid content in different regions of the brain, except for sulfatide levels (Han et al. 2003). Similarly, Sharman et al. (2010) found only minor differences in PE, SM, TG and cholesterol levels when assessing the total brain lipid levels in mice homozygous for *ε2, ε3* and *ε4* alleles. However, it must be taken into account that most of these slight differences were observed in 1-year-old mice but not in 2-months-old mice, which were also included in the study. Considering that we focused on differences in early life while the other two studies focused on the adult period, we hypothesize that the required lipid input during development may be increasing the differences between groups. Nevertheless, once this period is over, the lipid levels may stabilize until other challenging situations requiring regeneration such as trauma or infection appear.

Taking into account that this study focuses on the developmental period, a different maturation pattern might be contributing to the observed differences between genotypes, suggesting an earlier maturation of apoE4-TR mice. Previous studies found apoE3-TR mice presenting a delayed eye opening during the developmental period compared to apoE4-TR mice and a different expression of cholinergic elements in the brain (Basaure et al. 2018). Furthermore, a higher presence of age-related species in apoE4-TR mice microbiota were observed during the postnatal period (Guardia-Escote et al. 2020). An earlier onset of development was also found in young children carrying the *ɛ4* allele, suggesting they have a cognitive advantage over non-carriers (Remer et al. 2020). Chen et al. (2021) observed that the metabolomic signature was different in the neonatal and adult brain in mice. More specifically, PC and TAG were found at higher levels in adult brains (Chen et al. 2021). These findings are in line with the results of the current study, which show higher concentrations of these lipid families in the brains of apoE4-TR mice compared to the other groups.

This hypothesis could also explain the differences observed between treatments. More specifically, the treatment with CPF was observed to have a significant effect on ChoE 18:3 and several TG species, being CPF-treated group the one showing a lower concentration of lipids compared to control mice. It has been previously reported that postnatal CPF exposure delays physical maturation and alters the expression of cholinergic elements in the brain such as choline acetyltransferase, the α4-subunit and the α7 nicotinic acetylcholine receptors (nAChR) (Basaure et al. 2018). It should also be taken into account that development is a vulnerable period for toxic exposure, since the levels of the detoxification enzyme PON1 are lower at birth (Marsillach et al. 2016). Additionally, studies have reported that CPF exposure can decrease PON1 levels in both plasma and the brain, presumably due to associated liver damage, thereby leading to alterations in the plasma lipid profile (Deveci and Karapehlivan 2018). Consistent with these findings, a systematic review also highlighted liver damage as a significant factor contributing to the observed association between CPF exposure and changes in the blood lipid profile in rats and fish (Farkhondeh et al. 2022).

ChoE is a metabolite of cholesterol generated by lecithin–cholesterol acyltransferase (LCAT) in response to the excess of cholesterol in the brain. The local synthesis of cholesterol in the brain undergoes a rapid increase within the first three postnatal weeks, along with a transitory increase in ChoE, which will subsequently decrease over time (Kinney et al. 1994; Dietschy 2009; Phillips et al. 2020). Cholesterol and ChoE also conform lipid rafts, cholesterol-rich membrane domains involved in such functions as membrane signaling or axonal development, the disruption of which has been considered to be an early marker for neurodegenerative diseases (Ooi et al. 2021). The finding of higher ChoE levels in apoE4-TR mice suggests brain maturation is faster in those groups than in apoE3-TR mice. However, it has been reported that the *APOE4* genotype presents lower levels of LCAT (Mahley 2016), which would imply a higher basal level of cholesterol. In fact, a recent study in vitro found that the synthesis of cholesterol by astrocytes in the *APOE4* genotype was higher than in *APOE3* (Lee et al. 2021)*.* Despite the fact that the higher levels of ChoE were observed early in life, it must be taken into account that if maintained over time, high levels of ChoE can alter neuronal function, and they have been associated with conditions such as AD, multiple sclerosis and brain injury (van der Kant et al. 2019; Phillips et al. 2020). On the other hand, CPF was able to modulate ChoE levels, and apoE4-TR mice responded differently to the pesticide compared to their counterparts. Further investigations are required to unravel the underlying mechanism.

DG is a second messenger with many functions in the cells. Its hydrolysis by DG lipase results in 2-arachidonoylglycerol, a molecule involved in endocannabinoid signaling (Reisenberg et al. 2012). We observed that DG levels were higher in apoE4-TR and C57BL/6 mice than in apoE3-TR mice. In turn, a recent study described differences in the levels of DG in the brain in 14 to 15-month-old apoE-TR mice: apoE4-TR mice presented lower DG levels than apoE3-TR mice in the entorhinal cortex and the primary visual cortex (Miranda et al. 2022). This finding and our results support the importance of age for this important signaling lipid, and suggest potential alterations in the brain of aged apoɛ4 carrier’s. We also found that apoE4-TR mice had high levels of LPC, a phospholipid linked to such neurodegenerative diseases as AD. Indeed, LPC has been related to inflammation, demyelination in the CNS and alterations in the microvasculature of the brain, which enhance pathways leading to apoptosis (Sun et al. 2009; Plemel et al. 2018; Liu et al. 2021). Even though the present study focuses on early-age mice and further studies are required to assess whether these observations are maintained over time, we cannot dismiss the potential link between higher LPC levels and apoɛ4 carriers.

Likewise, higher levels of PC and SM, both membrane choline-containing phospholipids, were observed in apoE4-TR mice. Choline is also the precursor of the neurotransmitter acetylcholine (ACh), a key element of the cholinergic system. Since differences in the cholinergic system have been previously described between apoɛ3 and apoɛ4 carriers (Basaure et al. 2018), it is not surprising to find that the genotype has a significant effect in this regard. More specifically, Basaure et al. (2018) reported differences in the gene expression of vesicular acetylcholine transporter (VAChT), α7 nAChR, acetylcholinesterase-S (AChE-S) and acetylcholinesterase-R (AChE-R) in the forebrain between apoE3- and apoE4-TR mice during development. Particularly, apoE3-TR mice showed higher levels of VAChT and AChE-S, whereas apoE4-TR mice presented higher levels of α7 nAChR and AChE-R (Basaure et al. 2018). All in all, and considering that SM increases during the first years of life in humans (Dawson 2015), we can yet again relate higher levels of SM with enhanced brain maturation in apoE4-TR mice. On the other hand, it should be highlighted that the genetic background can lead to differences in PC levels after postnatal CPF exposure. Unquestionably, C57BL/6 mice are the most sensitive to CPF effects, which triggered a decrease in the PC concentrations in the brain. Nevertheless, whereas apoE3-TR mice present an increase in two different PC species after exposure to the pesticide, the concentrations in apoE4-TR mice remained unaltered. These results clearly show that vulnerabilities to the pesticide depend on the *APOE* genotype. Interestingly, this has also been observed in 15-days-old mice exposed to CPF for the same period. The levels of some short-chain fatty acids (SCFA), in particular isovaleric and 4-methylvaleric acid, in the brain were altered in all genotypes except *APOE4* (Guardia-Escote et al. 2020).

Lastly, apoE4-TR mice presented higher levels of TG in the brain than those found in the other groups. Previous studies in human iPSC-derived astrocytes showed that the ones expressing *APOE4* can accumulate higher levels of TG than the ones expressing *APOE3* (Sienski et al. 2021). These can alter the lipid balance in the brain and modify the levels of hypothalamic feeding hormones, such as leptin, insulin or ghrelin. More specifically, TGs are involved in transporting these hormones through the BBB and, therefore, regulating feeding behavior (Rhea and Banks 2021). High levels of TG would inhibit leptin transport through the BBB and increase the appetite. However, apoE3-TR mice usually present higher body weight compared to apoE4-TR mice (Huebbe et al. 2015). Along the same lines, the decrease in the TG levels after exposure to CPF would not correlate with the obesogenic effects previously observed in preadipocytes in vitro (Blanco et al. 2020) or in vivo (Fang et al. 2018), suggesting that other modulatory factors are involved.

One of the limitations of this study is that it focuses only on males although the literature shows the importance of including both males and females. Moreover, in a previous study we found that sex plays an important role in the response to toxic insults due to differences in metabolic regulation between sexes and the isoforms of *APOE* (Guardia-Escote et al. 2021)*.* Another limitation worth mentioning is that the brain samples were obtained 4 h after exposure to the pesticide, so the observed changes may be permanent or transitory. Also, we studied the whole brain whereas it has been shown that different brain areas have different lipid compositions when studied independently (Zhang et al. 1996). Hence, further follow–up investigations are required on both male and female mice at different time-points.

To sum up, the current results provide information about the lipid profile in the brain of male mice in the late postnatal period, and the potential neurotoxic effects of postnatal exposure to CPF and its interaction with the *APOE* genetic background. The *APOE* genotype plays an important role in the regulation of lipid composition, with apoE4-TR mice showing a different profile in most cases. These differences can be explained by a different maturation pattern between genotypes. Similarly, exposure to CPF can also modulate several lipid species by decreasing their concentration in the brain. The interactions between the genotype and the treatment clearly show different vulnerabilities to the neurotoxic effect of CPF depending on the apoE isoform. Altogether, these results show significant differences early in life, with potential implications for neurodevelopment and cognitive functioning.

## Data Availability

Data is still being exploded by the authors. However it will be available upon request.
